# Stop!

**Published:** 1916-10

**Authors:** 


### STOP!

So many cases have come under our observation recently where the contact points on the proximal surfaces of teeth and fillings have been ruined by cutting them away in the practice of so-called prophylaxis that it seems imperative to call a halt on such work in the most emphatic manner. The legitimate practice of prophylaxis does not contemplate any such desecration as this. In fact, the preservation of the normal contact between teeth is one of the prime requisites of prophylaxis and he who passes a disk or sandpaper strip between the contact points and cuts them down so as to narrow the mesio-distal width of the teeth and loosen up the contacts is not practicing prophylaxis in its truest sense, but is doing infinitely more harm than good. In the minds of some operators there is only the one idea of smoothness—everything else is negligible—and to make the enamel smooth they grind and trim and polish without discrimination, even to the contact points and on the occlusal surfaces. This is not prophylaxis—it is vandalism. To remove every last bit of salivary and serumal deposit from the teeth, to trim overhanging edges of fillings flush with the cavity margins, and then to polish the surfaces of the tooth and filling smooth and bright is legitimate prophylaxis, but it is not legitimate to so deform the tooth that the chief function for which the tooth was made is rendered impossible. The first result of cutting away the contacts is to permit food to lodge in the interproximal spaces, which makes the patient most uncomfortable. He can not masticate with the same degree of satisfaction as he could before he was given the prophylactic treatment, and he becomes impatient with dental service. The next result, if the difficulty is not remedied by restoring the contact, is to create pockets in the interproximal spaces and start the case on the rapid road to pyorrhea. Thus one of the chief ailments for which prophylaxis is supposed to be practiced is actually brought about by it when performed injudiciously.

What the profession most needs is breadth of vision and comprehensiveness of thought so that they may see and think beyond the narrow bounds of the immediate thing they are doing. There is something else besides making a tooth smooth. It is infinitely more important to maintain the normal function of mastication, and this can not be done by cutting away the contact points. In fact, be it said, the main factor in keeping the enamel polished smooth is the full process of mastication. The surface of no tooth is ever satisfactory unless wiped constantly by friction of the natural processes in the mouth, such as food in mastication, the friction of the lips, tongue or cheeks. We aid greatly in some instances by legitimate prophylactic measures, but we are never able to wholly compensate for loss of function as expressed in impaired masticating efficiency.

When prophylaxis is practiced let it be done conservatively and judiciously, and let us see no more of the havoc that is being wrought by deforming the teeth—Editorial in October *Review*.

